# Cysteines and Disulfide-Bridged Macrocyclic Mimics of Teixobactin Analogues and Their Antibacterial Activity Evaluation against Methicillin-Resistant *Staphylococcus Aureus* (MRSA)

**DOI:** 10.3390/pharmaceutics10040183

**Published:** 2018-10-11

**Authors:** Ruba Malkawi, Abhishek Iyer, Anish Parmar, Daniel G. Lloyd, Eunice Tze Leng Goh, Edward J. Taylor, Sarir Sarmad, Annemieke Madder, Rajamani Lakshminarayanan, Ishwar Singh

**Affiliations:** 1 School of Pharmacy, Joseph Banks Laboratories, University of Lincoln, Green Lane, Lincoln LN6 7DL, UK; 16657045@students.lincoln.ac.uk (R.M.); Abhishek.Iyer@UGent.be (A.I.); aparmar@lincoln.ac.uk (A.P.); 2 Organic and Biomimetic Chemistry Research Group, Department of Organic and Macromolecular Chemistry, Ghent University, Krijgslaan 281 (S4), B-9000 Ghent, Belgium; annemieke.madder@ugent.be; 3 School of Life Sciences, Joseph Banks Laboratories, University of Lincoln, Green Lane, Lincoln LN6 7DL, UK; dlloyd@lincoln.ac.uk (D.G.L.); etaylor@lincoln.ac.uk (E.J.T.); 4 Singapore Eye Research Institute, The Academia, Discovery Tower Level 6, 20 College Road, Singapore 169857, Singapore; eunice.goh.t.l@seri.com.sg (E.T.L.G.); lakshminarayanan.rajamani@seri.com.sg (R.L.); 5 School of Chemistry, Joseph Banks Laboratories, University of Lincoln, Green Lane, Lincoln LN6 7DL, UK; SSarmad@lincoln.ac.uk

**Keywords:** teixobactin, MRSA, disulfide bridge

## Abstract

Teixobactin is a highly potent cyclic depsipeptide which kills a broad range of multi-drug resistant, Gram-positive bacteria, such as Methicillin-resistant *Staphylococcus aureus* (MRSA) without detectable resistance. In this work, we describe the design and rapid synthesis of novel teixobactin analogues containing two cysteine moieties, and the corresponding disulfide-bridged cyclic analogues. These analogues differ from previously reported analogues, such as an Arg_10_-teixobactin, in terms of their macrocyclic ring size, and feature a disulfide bridge instead of an ester linkage. The new teixobactin analogues were screened against Methicillin-resistant *Staphylococcus aureus* and Methicillin-sensitive *Staphylococcus aureus*. Interestingly, one teixobactin analogue containing all l-amino acid building blocks showed antibacterial activity against MRSA for the first time. Our data indicates that macrocyclisation of teixobactin analogues with disulfide bridging is important for improved antibacterial activity. In our work, we have demonstrated the unprecedented use of a disulfide bridge in constructing the macrocyclic ring of teixobactin analogues.

## 1. Introduction

**Figure 1:**
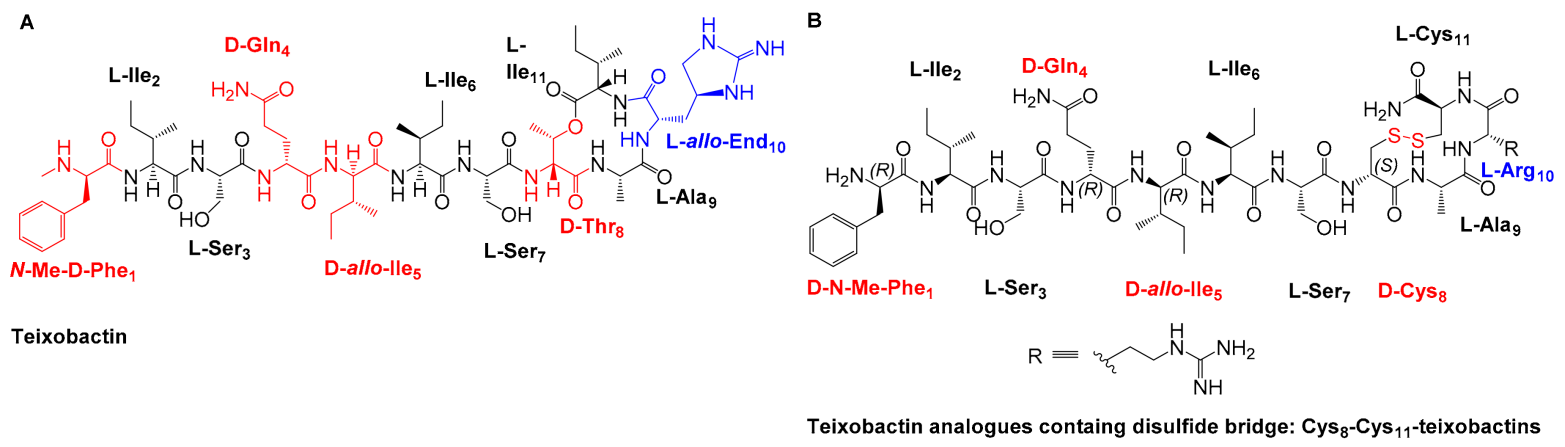
(**A**) Teixobactin, (**B**) Cys_8_–Cys_11_ disulfide-teixobactins.

Antimicrobial resistance (AMR) is predicted to cause 10 million deaths every year, and US $100 trillion in economic damages by 2050 [1]. Current antibiotics have proven to be ineffective against the recent surge of drug-resistant bacteria. Furthermore, the discovery and development of new antibiotics has been considerably slower than increase in bacterial resistance. Thus, there is a constant need to develop new and potent antibacterial compounds. The recently discovered natural product, teixobactin (Figure 1A) has shown highly potent activity against major, clinically relevant, Gram-positive bacteria, including resistant bacterial strains such as *Enterococcus spp.* (vancomycin-resistant enterococci, VRE), Methicillin-resistant *Staphylococcus aureus* (MRSA), and *Mycobacterium tuberculosis* [2]. The nonribosomal cyclic undecapeptide, teixobactin, comprises of four d-amino acids—namely, *N*-Me-d-Phe_1_, d-Gln_4_, d-*allo*-Ile_5_, and d-Thr_8_, and the rare l-*allo*-enduracididine amino acid. Teixobactin kills a number of Gram-positive bacteria without detectable resistance. Furthermore, it is less prone to developing resistance because it operates by at least two known unique modes of action. Teixobactin binds to lipid II (precursor of peptidoglycan), which is a highly conserved pyrophosphate motif of multiple bacterial cell wall substrates and lipid III (a precursor of cell wall teichoic acid) [2]. Previous publications on teixobactin describe the total syntheses of teixobactin [3,4] and its analogues [5,6,7], as well as their biological activities. Our group [6] and others [5,7] have reported the synthesis of Arg_10_-teixobactin by replacing the synthetically challenging l-*allo*-enduracididine amino acid occurring in native texiobactin at position 10 with arginine. The first structure-activity relationships (SAR) of Arg_10_-teixobactin and the importance of the four d-amino acids for antibacterial activity was also described by our research group [6]. Subsequently, we also defined the 3D molecular structures of teixobactin analogues using NMR [8]. The disordered structure of teixobactin analogues was found to be crucial for their biological activity, whereas the d-amino acids were found to be important for maintaining the disordered structure [8]. The crystal structure of a truncated teixobactin analogue was reported by Yang et al., and showed the Ile_6_ and Ile_11_ to be closely placed together through space [7]. 

Yang et al. [7] also reported the minimum pharmacophore of teixobactin, while Albericio et al. [9] reported on a lysine scan of Arg_10_-teixobactin. We further reported on the design and synthesis of potent teixobactin analogues against MRSA through the replacement of l-*allo*-enduracididine, showing that l-*allo*-enduracididine can be designed out and analogues can be made to realise the therapeutic potential of teixobactins [10,11].

To further improve access to highly potent teixobactin analogues, we also strategically replaced the synthetically challenging enduracididine with commercially available hydrophobic residues, such as leucine and isoleucine [12]. It was shown that Leu_10_-teixobactin and Ile_10_-teixobactin displayed activity against MRSA to the same extent as teixobactin. Brimble et al. have also reported on the synthesis and antibacterial activity of teixobactin analogues by replacing l-*allo*-enduracididine with isosteres [13]. Furthermore, teixobactin analogues with lipid tails were reported by Jamieson et al. [14] and recently we described highly potent teixobactin analogues containing proteogenic amino acids at position 10 and their antibacterial activity in vitro and in vivo [15]. As an alternative strategy, we were interested in exploring cysteines and disulfide-briged macrocyclic mimics of teixobactin analogues.

Cysteine-rich antimicrobial peptides containing multiple disulfide bonds were isolated from plants [16] and animals [17,18]. These peptides have shown protective effects in the living host against anti-bacterial infections [19]. In certain instances, disulfide bonds have been engineered to study the structure-activity relationships (SAR) of antimicrobial peptides, which makes them an interesting tool for SAR studies [20]. Furthermore, the easy introduction of cysteines via commercially available building blocks and mild conditions for synthesis of disulfide bonds [21] allow for rapid and cost-effective macrocyclization. In this study, we describe simplified teixobactin analogues by introducing cysteines at specific positions (d-Thr_8_ and Ile_11_) and subsequently constructing the macrocyclic ring using disulfide bridges to better understand the structure-activity relationship of teixobactin analogues (Figure 1B). This will also allow us to understand how the core ring size of teixobactin analogues (similar ring size: 14 atoms, teixobactin: 13 atoms) impacts its antibacterial activity.

Here, we report the design, rapid synthesis, and antibacterial activity evaluations against MRSA of eight new teixobactin analogues containing cysteines and the unprecedented use of a disulfide bridge in constructing the macrocyclic ring of teixobactin.

## 2. Materials and Methods

### 2.1. Materials

Fmoc-d-Ile-OH, Fmoc-d-Thr(Trt)-OH, and oxyma pure were purchased from Merck Millipore (Watford, UK). All l-amino acids, 1-[Bis(dimethylamino)methylene]-1H-1,2,3-triazolo[4,5-b]pyridinium 3-oxid hexafluorophosphate (HATU), Fmoc-d-Gln(Trt)-OH, Boc-d-*N*methylphenyl-OH, Fmoc-d-Cys-OH, Fmoc-l-Cys-OH, Fmoc-Glu(OAll)-OH, Diisoproplycarbodiimide, and Triisopropylsilane were purchased from Fluorochem, Hadfield, UK. The protecting groups for the amino acids are Pbf for Arg and Trt for Gln, unless specified otherwise. Diisopropylethylamine, supplied as extra-dry, redistilled, and 99.5% pure, was purchased from Sigma Aldrich (Gillingham, UK). Peptide-synthesis grade Dimmethylformamide (DMF) and Trifluoroacetic acid (TFA) was purchased from Rathburn chemicals (Walkerburn, UK). Diethyl ether, i-PrOH, MeOH (HPLC grade), and Acetonitrile (HPLC grade) were purchased from Fisher Scientific (Loughborough, UK). Water with the Milli-Q grade standard was obtained in-house from an ELGA Purelab Flex system. Rink amide Chemmatrix resin (manufacturer’s loading = 0.49 mmol/g) was obtained from Biotage, Uppsala, Sweden. All chemicals were used without further purification. 

### 2.2. General Methods

All peptides were analyzed on a Thermo Scientific Dionex Ultimate 3000 HPLC (Thermo Scientific, Hemel Hempstead, UK) equipped with a Phenomenex Gemini NX C18 110 Å (150 × 4.6 mm) (Phenomenex, Macclesfield, UK)column, using the following buffer systems: A: 0.1% HCOOH in milliQ water. B: ACN using a flow rate of 1 mL/min. The column was flushed with 95% A for 5 min prior to an injection and was flushed for 5 min with 95% B and 5% A after the run was finished. Peptides were dissolved in (1:1) 0.1% HCOOH buffer in water and acetonitrile (ACN) and analyzed using the following gradient: 95% A for 2 min; 5–95% B for 25 min; 95% B for 5 min; and 5% A for 4 min. Peptides were dissolved in 0.1% HCOOH buffer in water and in ACN (10–30% ACN), and purified using the same gradient as mentioned above, on a Thermo Scientific Dionex Ultimate 3000 HPLC with a flow rate of 5 mL/min using a Phenomenex Gemini NX C18 110 Å (150 × 10 mm) semi-prep column. ESIMS spectra were recorded on a Thermo Scientific Mass Spectrometer (TSQ Endura, triple quad) in the positive electrospray ion mode. 

### 2.3. Synthesis of Teixobactin Analogue 3

**Scheme 1:**
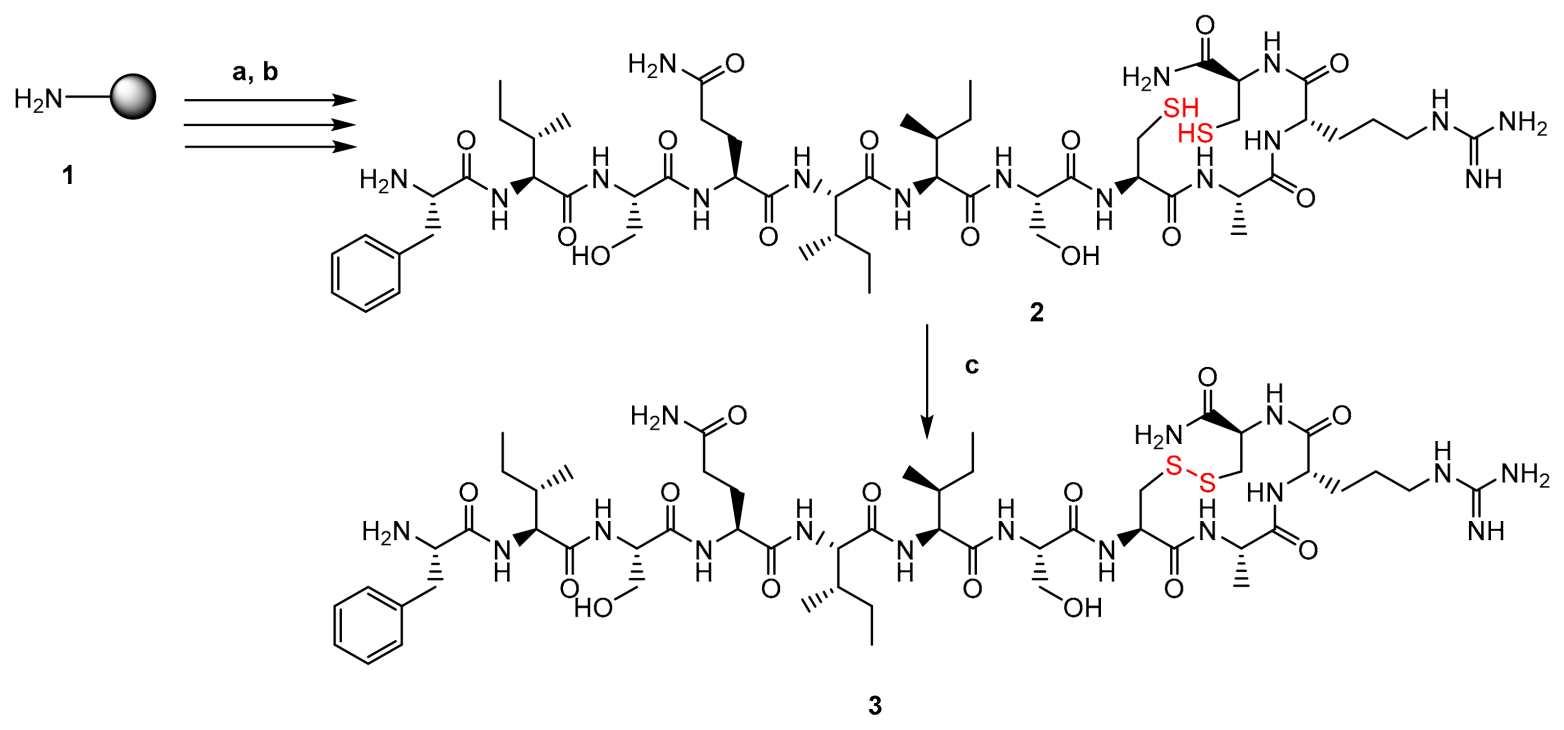
Synthesis of analogue **1**, **2**, and **3**: **a**. Fmoc-AA(PG)-OH (AA = amino acid, PG = protecting group), DIC/Oxyma microwave couplings, followed by 20% piperidine in Dimmethylformamide (DMF). **b**. TFA:TIS:H_2_O = 95:2.5:2.5, 1 h. **c**. DMSO:miliQ water = 1:3 (peptide concentration 1 mM), 12 h.

Teixobactin analogue 3 was synthesized as described in Scheme 1. (step **a**) Commercially available Rink amide Chemmatrix resin (manufacture’s loading 0.49 mmol, 204 mg, 0.1 mmol) resin was swelled in DMF in a reactor. To this resin all the amino acids in the sequence were coupled by the use of 4 equiv of amino acids, 4 equiv of DIC/Oxyma in a microwave peptide synthesiser (Biotage Initiator + Alstra). The coupling time was 5 min at 70 °C. The deprotection was done using 20% of Piperidine in DMF for 3 min at 70 °C, and then for 10 min at room temperature. (step **b**) After building the linear peptide **2**, full cleavage was achieved using TFA/TIS/H2O = 95:2.5:2.5% by being stirred for 1 h. The peptide was filtrated into cold diethyl ether (−20 °C) and the precipitate was centrifuged 3 × 10 min at 7000 rpm to obtain crude peptide. (step **c**) The crude peptide **2** was cyclized using DMSO/MQ water = 3:1 while maintaining a peptide concentration of 1 mM, and was stirred for 12 h at room temperature. The reaction mixture was purified by using semi-prep RP-HPLC (Figures S1, S3, S5, S7, S9, S11, S13 and S15 in Supporting Information) using the protocols described in the general methods. All the pure fractions were combined together and lyophilized to obtain a white solid. All the teixobactin analogues were purified to ~95% purity, as indicated by HPLC (Figures S1, S3, S5, S7, S9, S11, S13 and S15 in Supporting Information). All the peptides were synthesized using the method described above for teixobactin analogue **3**, and isolated yields were in the range of 15–25%. All teixobactin analogues were characterized by ESIMS in positive mode (Table S1, Figures S2, S4, S6, S8, S10, S12, S14 and S16).

### 2.4. Antibacterial Screening 

For minimum inhibitory concentration (MIC) assays, all peptides were dissolved in DMSO containing 0.002% polysorbate 80. Bacteria was grown in Mueller Hinton broth (Oxoid) in triplicate. All incubations were at 37 °C. Dilutions were carried out in triplicate, and 100 µL of autoclaved Mueller Hinton broth was added to wells 2–11 in a 96-well plate. 200 µL of the peptide was added to well one at a concentration of 128 µg/mL. 100 µL of peptide in well one was taken up and pipetted into well two. The mixture was then mixed via pipetting before 100 µL was taken up and pipetted into well three. This process was repeated up to well 10. Once the peptide was added to well 10, 100 µL was taken up and then discarded, ensuring that well 11 had no peptide present. Thus, the concentrations (in µg/mL) were: 128, 64, 32, 16, 8, 4, 2, 1, 0.5, 0.25, and 0 (no peptide present). Each well was then inoculated with 100 µL of bacteria that had been diluted to an OD 600 nm of 0.1. This was repeated three times. The 96-well plates were then incubated at 37 °C for 24 h. The MIC was determined to be the lowest concentration at which there was no growth visible.

## 3. Results and Discussion

### 3.1. Design and Synthesis 

In our major ongoing project to develop simplified teixobactin analogues against multi-drug resistant bacteria which are easily synthetically accessible, we were particularly interested in understanding the role of the ester bond and the ring size of the teixobactin macrocycle. To test this, we selected cysteines to replace d-Thr_8_ and Ile_11_, and a disulfide bridge instead of the ester linkage in the macrocyclic ring of teixobactin. This replacement allows for the synthesis of an analogue with a larger macrocyclic ring (14 atoms) similar to the native teixobactin macrocycle (13 atoms). The use of a disulfide bridge in teixobactin analogues allows the incorporation of all amino acids in an automated manner from commercially available building blocks. Moreover, the macrocyclization can be achieved in solutions using mild conditions, such as air oxidation. 

**Figure 2:**
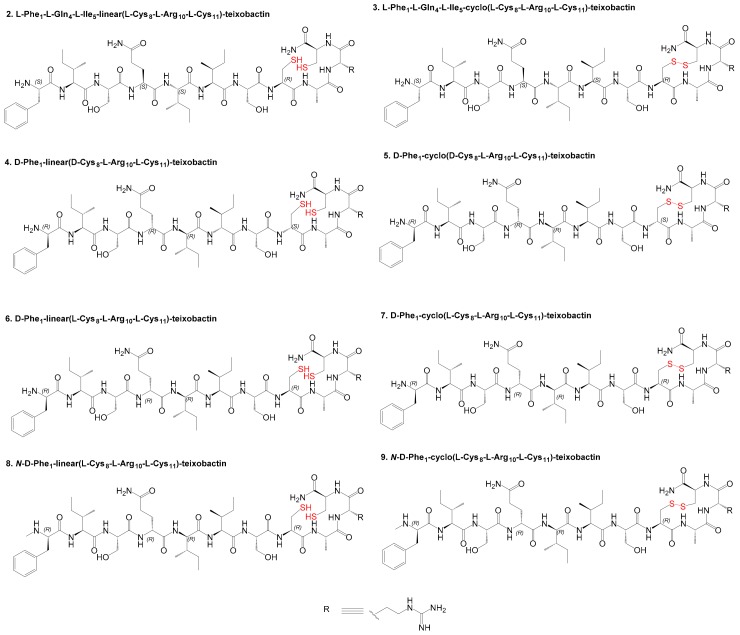
Structure of teixobactin analogues, **2**–**9**.

Teixobactin contains four d-amino acids (*N*-Me-d-Phe_1_, d-Gln_4_, d-*allo*-Ile_5_ and d-Thr_8_). To test the feasibility of the synthesis of teixobactin analogues containing a disulfide bridge, we selected all l-amino acids for cost-effectiveness. The synthesis of all teixobactin analogues **2**–**9** (Figure 2) were achieved successfully, as described in Scheme 1. After establishing the synthesis of teixobactin analogues with l-amino acids, we turned our attention to the synthesis of teixobactin analogues (**4**,**5**) with four d-amino acids similar to teixobactin. Biological evaluation of these analogues would facilitate an understanding of their role, as well as a comparison with teixobactin analogues containing l-amino acids (**2**,**3**). d-amino acids of teixobactin analogues are reported to be important for their biological activity. To test whether this was also true with cysteines containing teixobactin analogues, we synthesized analogues (**6**,**7**). The analogues (**8**,**9**) were synthesized to evaluate the role methyl group at d-phenylalanine_1_ in antibacterial activity.

The synthesis of teixobactin analogue **3** started on Rink amide Chemmatrix resin, using an automated microwave peptide synthesizer. The coupling of all amino acid (AA) building blocks was achieved using four equivalents of AA and 4 equivalents of DIC/Oxyma at 70 °C for 5 min. Fmoc deprotection was performed using 20% piperidine in DMF (Scheme 1). On completion of the synthesis, the peptide was released and deprotected from resin in a single step using TFA:TIS:H_2_O. The crude teixobactin analogue **2** was cyclized in DMSO and miliQ water using air oxidation to obtain analogue **3**. Analogue **3** was purified using reverse phase HPLC (Figure S1), and its identity was confirmed by ESI-MS (Figure S2). The isolated yields of teixobactin analogues were found to be 15–25%.

### 3.2. Antibacterial Studies

Antibacterial activity of the teixobactin analogues was assessed against Methicillin-resistant *Staphylococcus aureus* (MRSA ATCC 33591, MRSA ATCC 70069) and Methicillin-sensitive *Staphylococcus aureus*. The minimum inhibitory concentration was measured using the broth microdiluation method (see Experimental Section 2.4). Interestingly, the linear analogues **2** showed very low activity (MIC 128 µg/mL, Table 1) against MRSA ATCC 33591. The cyclisation of linear teixobactin analogues is reported to improve antibacterial activity [7]. The analogue **3** surprisingly showed 2–4 times higher activity (MIC 32–64 µg/mL) than analogue **2** against MRSA ATCC 33591, despite having all l-amino acids. These results are of interest since, thus far, there has been no activity reported for linear or cyclic analogues of teixobactin aganist MRSA containing all l-amino acids. Analogue **3** did not exhibit any activity against MRSA ATCC 70069 and Methicillin-sensitive *Staphylococcus aureus* up to the tested concentration of 128 µg/mL. Unexpectedly, the analogues **4**,**5** containing d-amino acids (d-Phe_1_, d-Gln_4_, d-*allo*-Ile_5_ and d-Cys_8_) have not shown any activity against MRSA and MSAA. This observation is contrary to previous findings where teixobactin analogues containing ester linkages and d-amino acids were reported to show good antibacterial activity [8]. Thus, our finding indicates that teixobactin analogues containing a disulfide bridge do not follow the structure activity trend of teixobactin analogues containing an ester linkage in the macrocycle. The analogues **6**,**7** and **8**,**9** containing three d-amino acids (d-Phe_1_ (for **6**,**7**), *N*-d-Phe_1_ (for **8**,**9**), d-Gln_4_, d-*allo*-Ile_5_ and l-Cys_8_) have not shown any antibacterial activity against MRSA and MSSA. The low activity (**3**) and lack of any antibacterial activity (**4**–**9**) of teixobactin analogues is most probably due to a larger ring size (14 atoms) and lack of hydrophobic contact between Ile_6_ and Ile_11_ [22] due to the replacement of Ile_11_ with cysteine in teixobactin analogues containing a disulfide bridge.

## 4. Conclusions

We have developed a rapid synthesis of new teixobactin analogues, either containing cysteines or a disulfide bridge. This synthesis uses the automated incorporation of all amino-acid building blocks. The teixobactin analogue containing l-amino-acid building blocks with a disulfide bridge showed antibacterial activity against MRSA. These results suggest that the cyclization of teixobactin analogues is important, but also that d-amino acids are not essential for antibacterial activity against MRSA. Moreover, we have demonstrated the unprecedented use of a disulfide bridge in constructing the macrocyclic ring of teixobactin analogues. Our work lays the foundation for rapid synthesis of further simplified teixobactin analogues containing cysteines and a disulfide bridge, and may provide molecules suitable for addressing the challenges from antimicrobial resistance. 

## Figures and Tables

**Table 1:** List of teixobactin analogues (**2**–**9**). MIC: Minimum Inhibitory Concentration in µg/mL; all the MIC runs in triplicate. (-) = Not evaluated.

Compound No.	MRSA ATCC 33591	MRSA ATCC 700699	S. aureus ATCC 29213
**2**	128	>128	>128
**3**	32–64	>128	>128
**4**	>128	>128	>128
**5**	>128	>128	>128
**6**	>128	-	>128
**7**	>128	-	>128
**8**	>128	-	>128
**9**	>128	-	>128
Vancomycin	-	2	4
Leu_10_-teixobactin^12^	0.25	0.25	0.062
Teixobactin^12^	0.25	-	-

## Supplementary Materials

The following are available online at http://www.mdpi.com/1999-4923/10/4/183/s1, Table S1: Compound number, name, chemical formula, exact mass and mass found for compounds 2–9. Figure S1: HPLC trace of purified teixobactin analogue **2** (gradient: 5–95% ACN in 25 min using. A: 0.1% HCOOH in water, B: ACN). Figure S2: ESI-MS of purified teixobactin analogue **2**. Exact mass calcd. For C_53_H_90_N_16_O_14_S_2_ = 1238.63, found M + H^+^ = 1239.62, M/2 + H^+^ = 620.38. Figure S3: HPLC trace of purified teixobactin analogue **3** (gradient: 5–95% ACN in 25 min using A: 0.1% HCOOH in water, B: ACN). Figure S4: ESI-MS of purified teixobactin analogue **3**. Exact mass calcd. For C_53_H_88_N_16_O_14_S_2_ = 1236.61, found M + H^+^ = 1237.72, M/2 + H^+^ = 619.47. Figure S5: HPLC trace of purified teixobactin analogue **4** (gradient: 5–95% ACN in 25 min using A: 0.1% HCOOH in water, B: ACN). Figure S6: ESI-MS of purified Teixobactin analogue **4**. Exact mass calcd. For C_53_H_90_N_16_O_14_S_2_ = 1238.63, found M + H^+^ = 1239.57, M/2 + H^+^ = 620.76. Figure S7: HPLC trace of purified teixobactin analogue **5** (gradient: 5–95% ACN in 25 min using A: 0.1% HCOOH in water, B: ACN). Figure S8: ESI-MS of purified teixobactin analogue **5**. Exact mass calcd. For C_53_H_88_N_16_O_14_S_2_ = 1236.61, found M + H^+^ = 1237.53, M/2 + H^+^ = 619.53. Figure S9: HPLC trace of purified teixobactin analogue **6** (gradient: 5–95% ACN in 25 min using A: 0.1% HCOOH in water, B: ACN). Figure S10: ESI-MS of purified teixobactin analogue **6**. Exact mass calcd. For C_53_H_90_N_16_O_14_S_2_ = 1238.63, found M + H^+^ = 1239.57, M/2 + H^+^ = 620.76. Figure S11: HPLC trace of purified teixobactin analogue **7** (gradient: 5–95% ACN in 25 min using A: 0.1% HCOOH in water, B: ACN). Figure S12: ESI-MS of purified teixobactin analogue **7**. Exact mass calcd. For C_53_H_88_N_16_O_14_S_2_ = 1236.61, found M + H^+^ = 1237.56, M/2 + H^+^ = 619.36. Figure S13: HPLC trace of purified teixobactin analogue **8** (gradient: 5–95% ACN in 25 min using A: 0.1% HCOOH in water, B: ACN). Figure S14: ESI-MS of purified teixobactin analogue **8**. Exact mass calcd. For C_54_H_92_N_16_O_14_S_2_ = 1252.64, found M + H^+^ = 1253.64, M/2 + H^+^ = 627.44. Figure S15: HPLC trace of purified teixobactin analogue **9** (gradient: 5–95% ACN in 25 min using A: 0.1% HCOOH in water, B: ACN). Figure S16. ESI-MS of purified teixobactin analogue **9**. Exact mass calcd. For C_54_H_90_N_16_O_14_S_2_ = 1250.63, found M + H^+^ = 1251.63, M/2 + H^+^ = 626.24.
