# Supplementary material for: Cysteines and Disulfide-Bridged Macrocyclic Mimics of Teixobactin Analogues and Their Antibacterial Activity Evaluation against Methicillin-Resistant Staphylococcus Aureus (MRSA)
Source: Pharmaceutics. 2018 Oct 11;10(4):183. doi: 10.3390/pharmaceutics10040183 (PMC6321233; doi:10.3390/pharmaceutics10040183)
Supplement: Supplementary file 1 [file pharmaceutics-10-00183-s001.pdf]

pharmaceutics

MDPI

Supplementary Materials: Cysteines and Disulfide-Bridged Macrocyclic Mimics of Teixobactin Analogues and Their Antibacterial Activity Evaluation against Methicillin-Resistant *Staphylococcus Aureus* (MRSA)

Ruba Malkawi, Abhishek Iyer, Anish Parmar, Daniel G. Lloyd, Eunice Tze Leng Goh, Edward J. Taylor, Sarir Sarmad, Annemieke Madder, Rajamani Lakshminarayanan, and Ishwar Singh

Table S1. Compound number, name, chemical formula, exact mass and mass found for compounds **2–9.**

Coumpound Number	Name	Chemical Formula	Mass Calcd (Da)	Mass Obsd (Da)
2	L-Phe1-L-Gln4-L-Ile5-linear(L-Cys8-L-Arg10-L-Cys11)-teixobactin	$C_{53}H_{90}N_{16}O_{14}S_2$	1239.63	1239.62
3	L-Phe1-L-Gln4-L-Ile5-cyclo(L-Cys8-L-Arg10-L-Cys11)-teixobactin	$C_{53}H_{88}N_{16}O_{14}S_2$	1237.61	1237.72
4	D-Phe1-linear(D-Cys8-L-Arg10-L-Cys11)-teixobactin	$C_{53}H_{90}N_{16}O_{14}S_2$	1239.63	1239.62
5	D-Phe1-cyclo(D-Cys8-L-Arg10-L-Cys11)-teixobactin	$C_{53}H_{88}N_{16}O_{14}S_2$	1237.61	1237.53
6	D-Phe1-linear(L-Cys8-L-Arg10-L-Cys11)-teixobactin	$C_{53}H_{90}N_{16}O_{14}S_2$	1239.63	1239.57
7	D-Phe1-cyclo(L-Cys8-L-Arg10-L-Cys11)-teixobactin	$C_{53}H_{88}N_{16}O_{14}S_2$	1237.61	1237.56
8	*N*-Me-D-Phe1-linear(L-Cys8-L-Arg10-L-Cys11)-teixobactin	$C_{54}H_{92}N_{16}O_{14}S_2$	1253.64	1253.64
9	*N*-Me-D-Phe1-cyclo(L-Cys8-L-Arg10-L-Cys11)-teixobactin	$C_{54}H_{90}N_{16}O_{14}S_2$	1251.63	1251.63

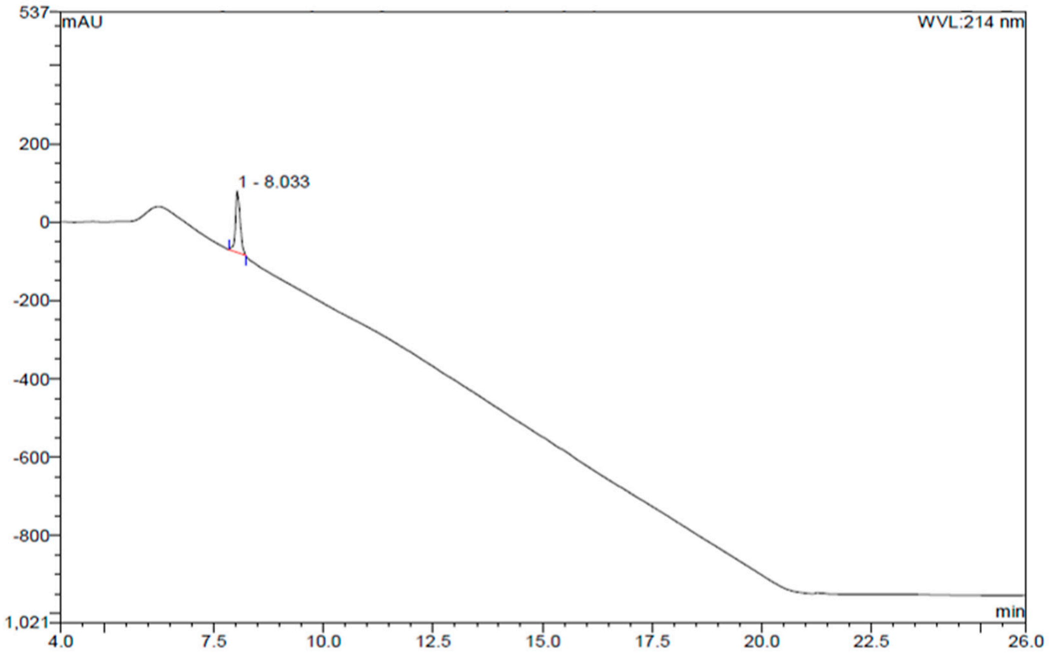

Figure S1. HPLC trace of purified teixobactin analogue **2** (gradient: 5–95% ACN in 25 min using. A: 0.1% HCOOH in water, B: ACN).

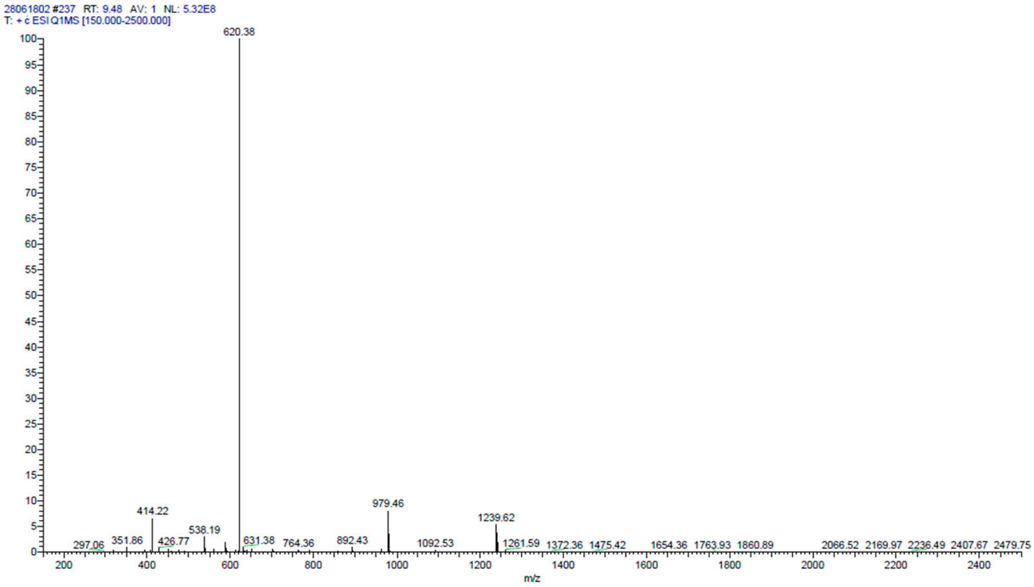

Figure S2. ESI-MS of purified teixobactin analogue **2**. Exact mass calcd. For $C_{53}H_{90}N_{16}O_{14}S_2$ = 1238.63, found M + H^+ = 1239.62, M/2 + H^+ = 620.38.

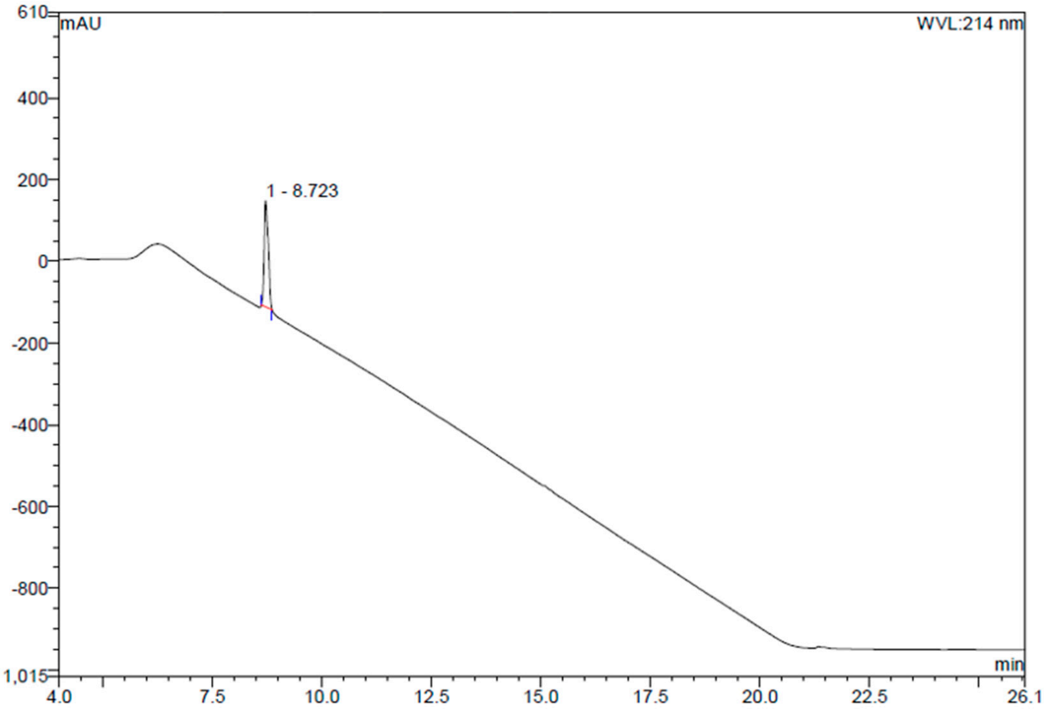

Figure S3. HPLC trace of purified teixobactin analogue **3** (gradient: 5–95% ACN in 25 min using A: 0.1% HCOOH in water, B: ACN).

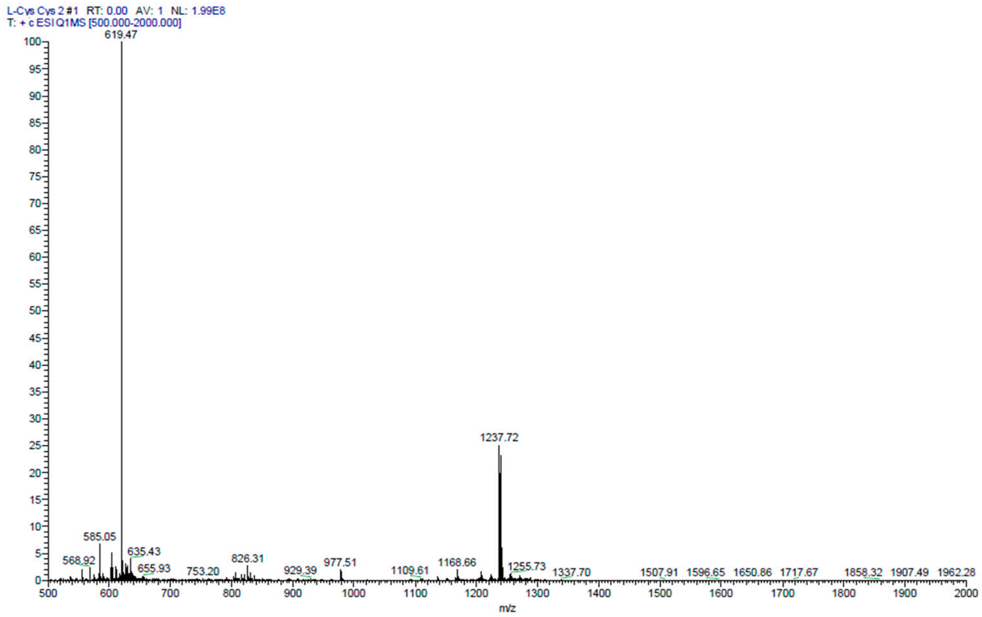

Figure S4. ESI-MS of purified teixobactin analogue **3**. Exact mass calcd. For $C_{53}H_{88}N_{16}O_{14}S_2$ = 1236.61, found M + H^+ = 1237.72, M/2 + H^+ = 619.47.

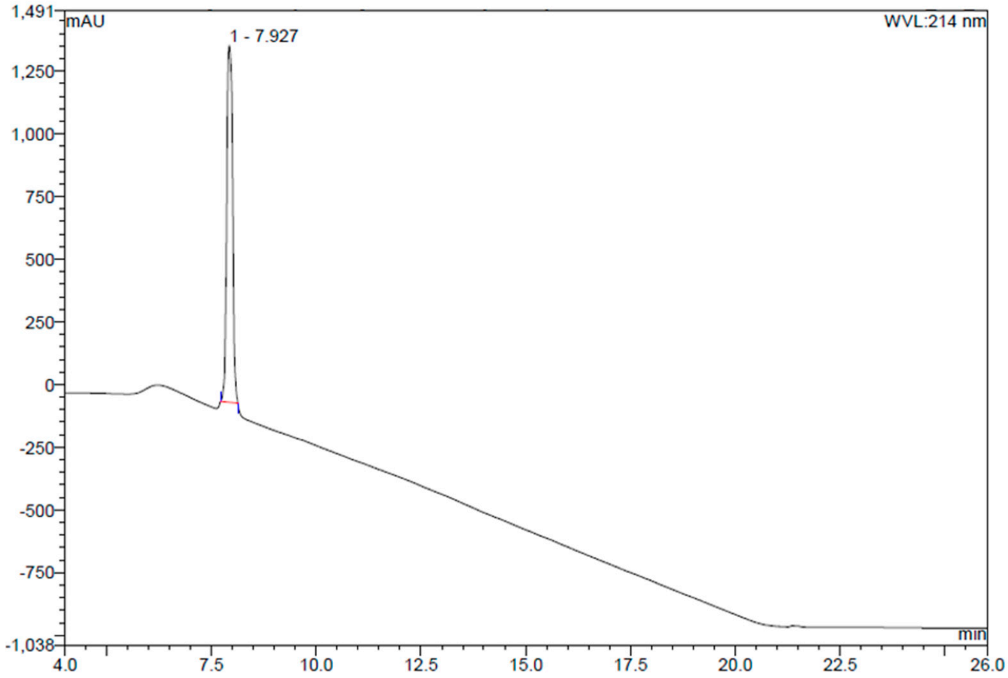

Figure S5. HPLC trace of purified teixobactin analogue **4** (gradient: 5–95% ACN in 25 min using A: 0.1% HCOOH in water, B: ACN).

pharmaceutics

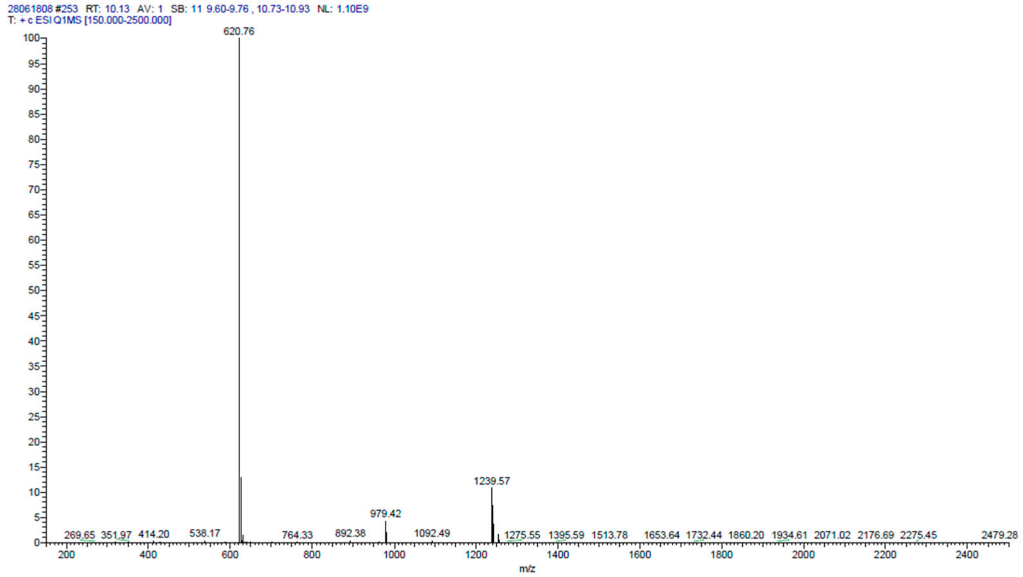

Figure S6. ESI-MS of purified Teixobactin analogue **4**. Exact mass calcd. For $C_{53}H_{90}N_{16}O_{14}S_2$ = 1238.63, found M + H⁺ = 1239.57, M/2 + H⁺ = 620.76.

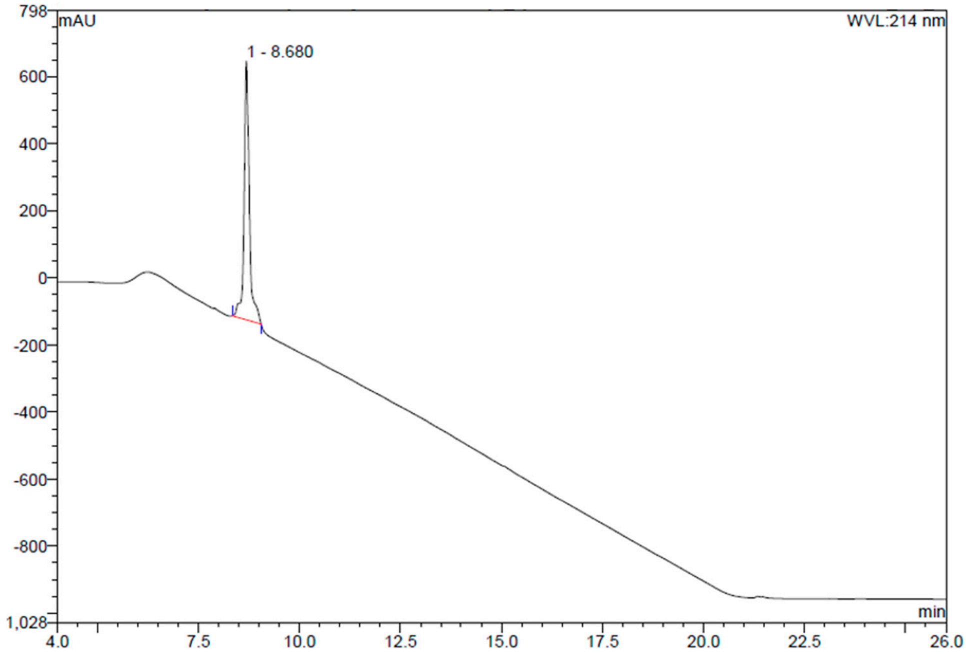

Figure S7. HPLC trace of purified teixobactin analogue **5** (gradient: 5–95% ACN in 25 min using A: 0.1% HCOOH in water, B: ACN).

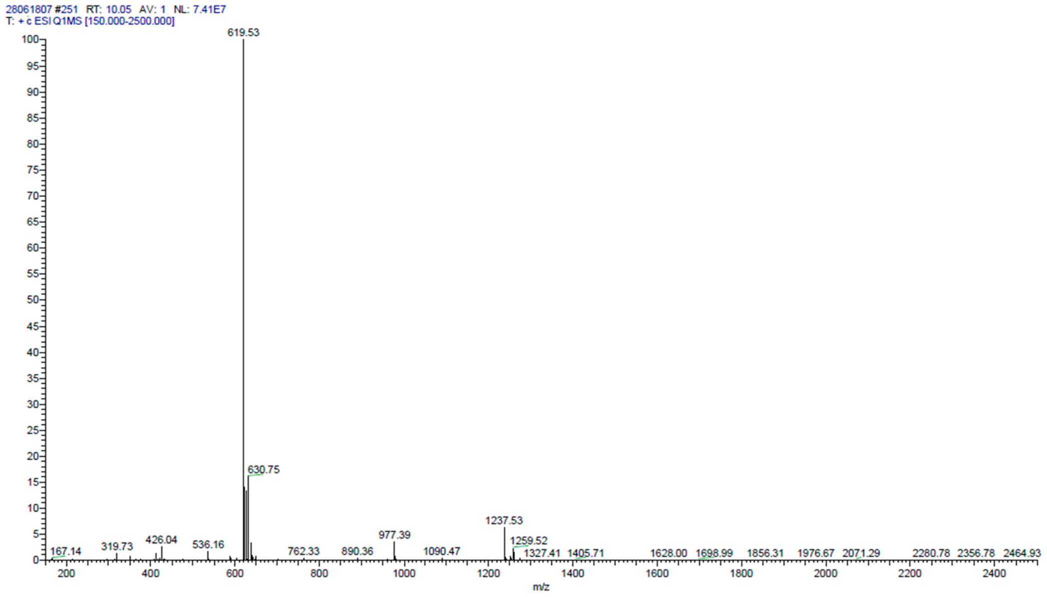

Figure S8. ESI-MS of purified teixobactin analogue **5**. Exact mass calcd. For $C_{53}H_{88}N_{16}O_{14}S_2$ = 1236.61, found M + H$^+$ = 1237.53, M/2 + H$^+$ = 619.53.

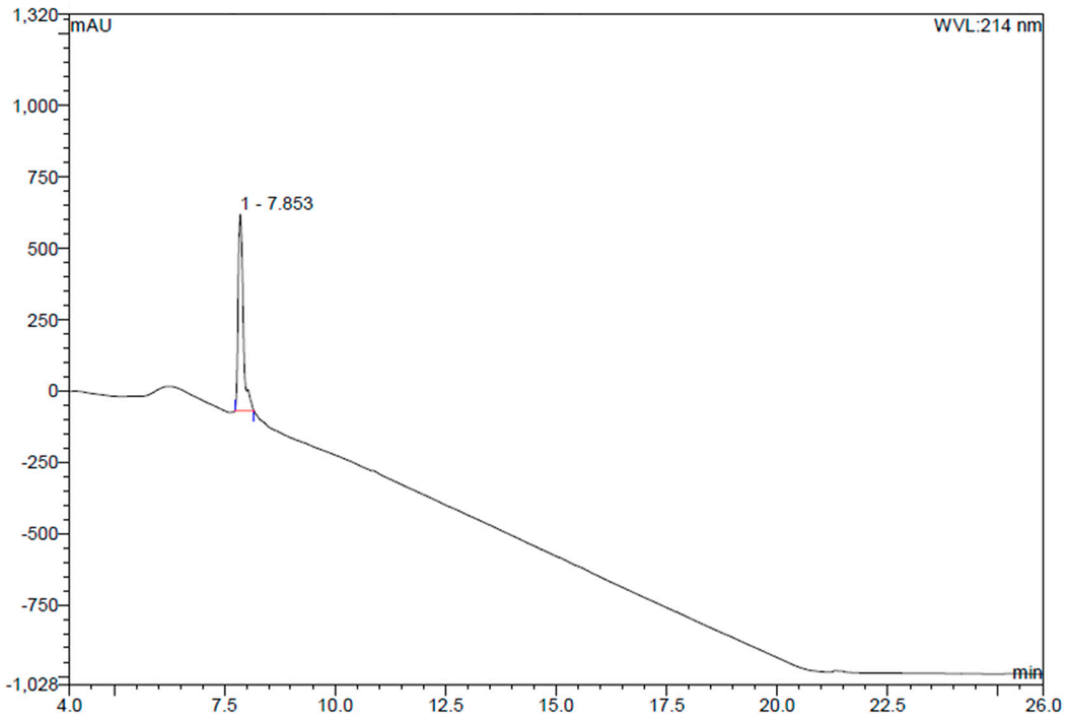

Figure S9. HPLC trace of purified teixobactin analogue **6** (gradient: 5–95% ACN in 25 min using A: 0.1% HCOOH in water, B: ACN).

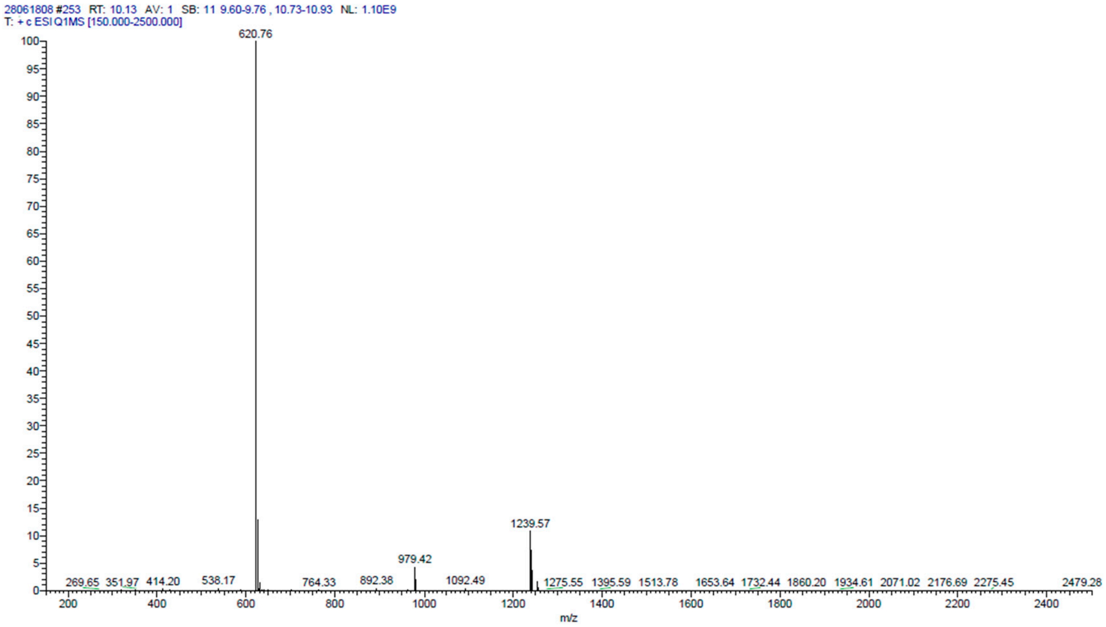

Figure S10. ESI-MS of purified teixobactin analogue **6**. Exact mass calcd. For $C_{53}H_{90}N_{16}O_{14}S_2$ = 1238.63, found M + H⁺ = 1239.57, M/2 + H⁺ = 620.76.

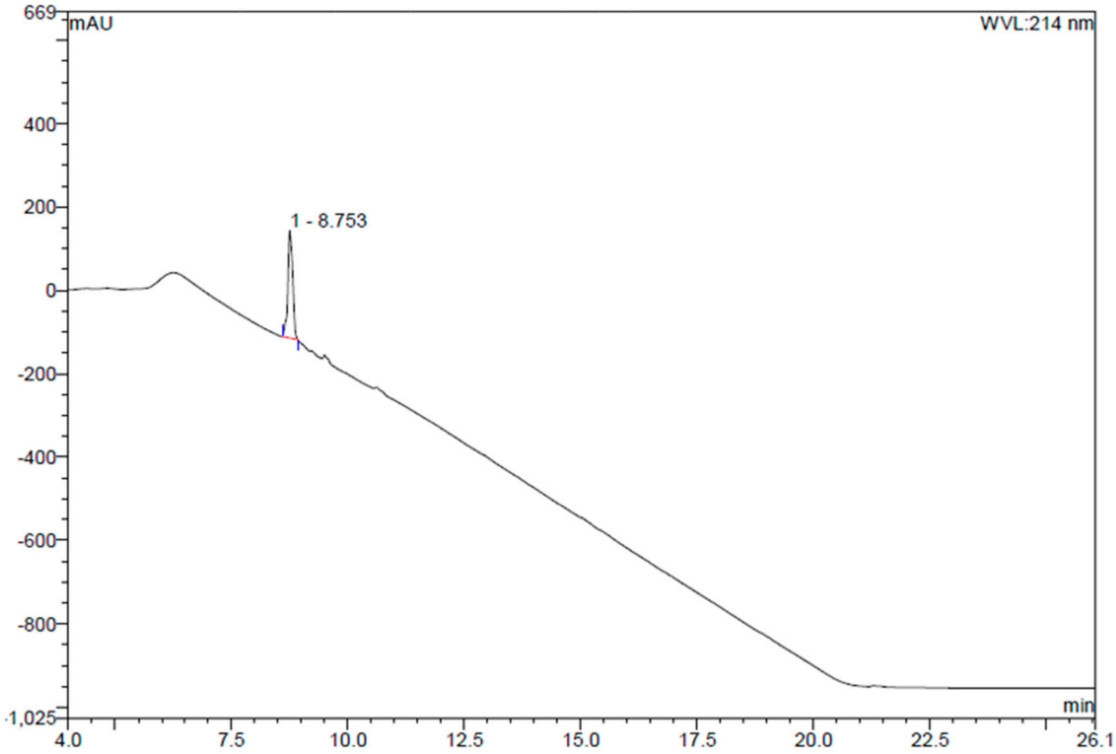

Figure S11. HPLC trace of purified teixobactin analogue **7** (gradient: 5–95% ACN in 25 min using A: 0.1% HCOOH in water, B: ACN).

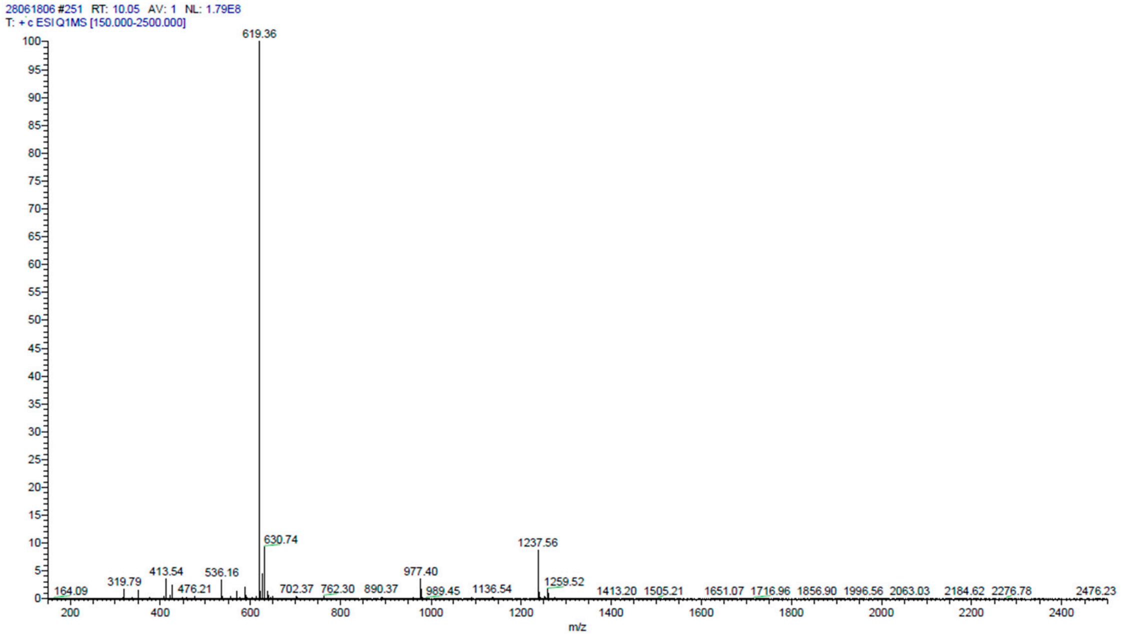

Figure S12. ESI-MS of purified teixobactin analogue **7**. Exact mass calcd. For $C_{53}H_{88}N_{16}O_{14}S_2$ = 1236.61, found M + H^+ = 1237.56, M/2 + H^+ = 619.36.

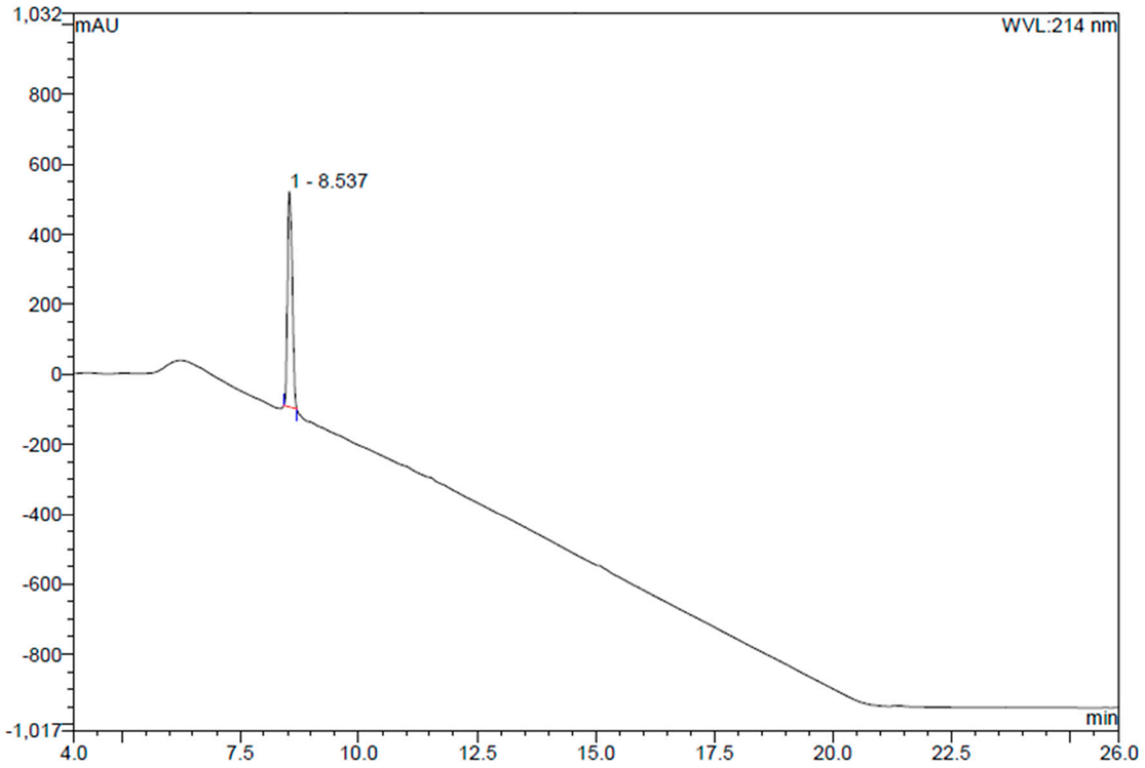

Figure S13. HPLC trace of purified teixobactin analogue **8** (gradient: 5–95% ACN in 25 min using A: 0.1% HCOOH in water, B: ACN).

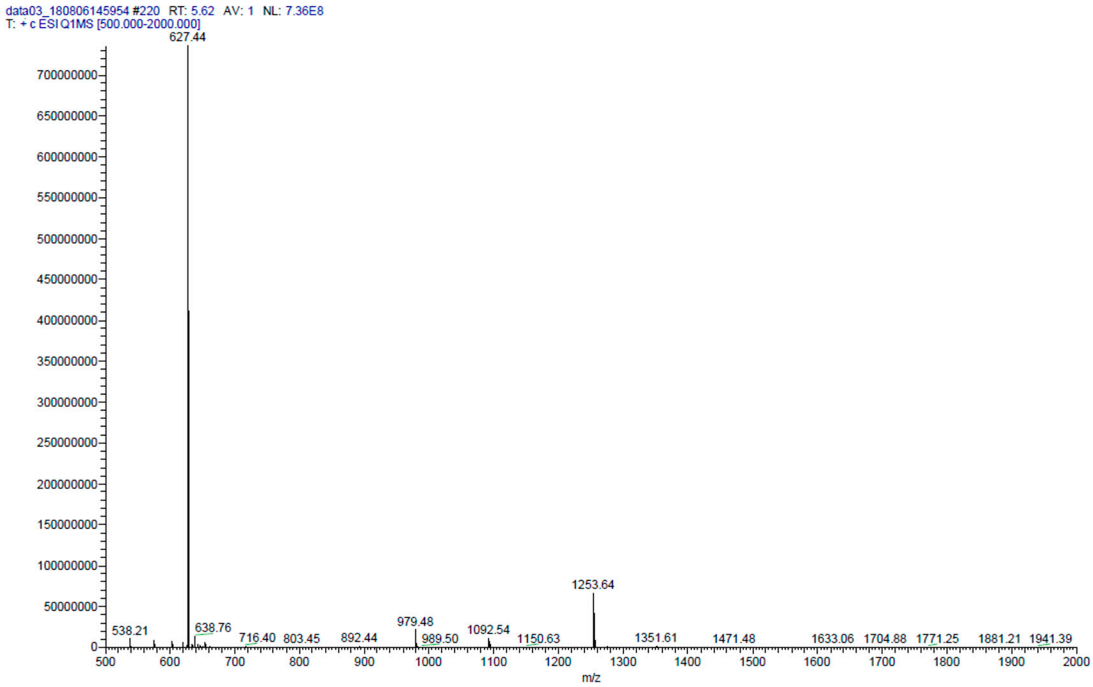

Figure S14. ESI-MS of purified teixobactin analogue **8**. Exact mass calcd. For $C_{54}H_{92}N_{16}O_{14}S_2$ = 1252.64, found M + H$^+$ = 1253.64, M/2 + H$^+$ = 627.44.

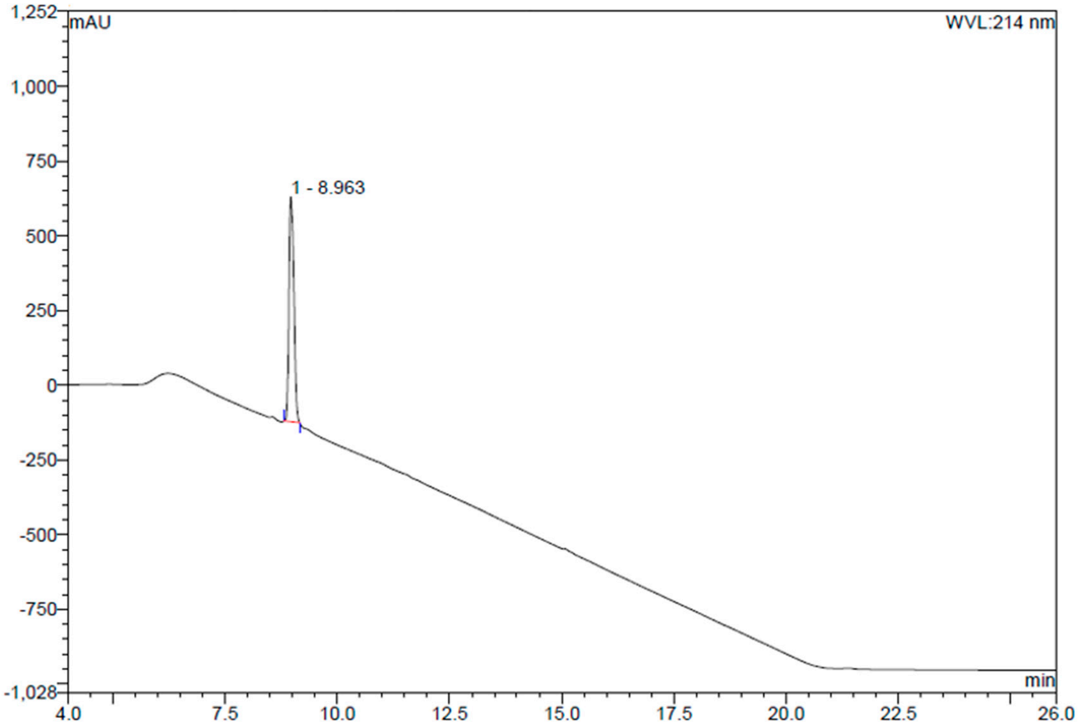

Figure S15. HPLC trace of purified teixobactin analogue **9** (gradient: 5–95% ACN in 25 min using A: 0.1% HCOOH in water, B: ACN).

pharmaceutics

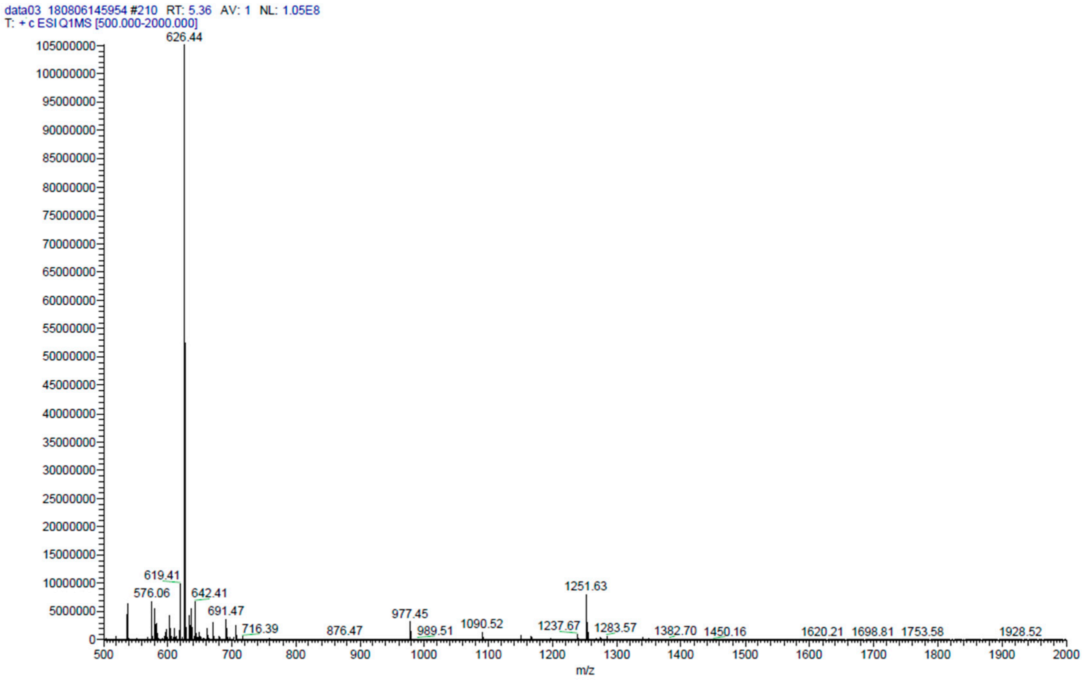

Figure S16. ESI-MS of purified teixobactin analogue 9. Exact mass calcd. For $C_{54}H_{90}N_{16}O_{14}S_2$ = 1250.63, found M + H⁺ = 1251.63, M/2 + H⁺ = 626.24.
